# Exploring the potential mechanisms of Pei Yuan Yi Fei decoction for non-small cell lung cancer through network pharmacology and experimental validation

**DOI:** 10.3389/fonc.2026.1938084

**Published:** 2026-10-19

**Authors:** Yingjia Zhou, Jia Wu, Mei Li, Xiaowei Jin, Shan Liu, Jian Yang, Hongmei Shen, Fan Li

**Affiliations:** 1 The Third Affiliated Hospital of Kunming Medical University, Yunnan Cancer Hospital, Peking University Cancer Hospital Yunnan, Integrated Traditional Chinese and Western Medicine Department, Xishan District, Kunming, Yunnan, China; 2 Kunming Medical University, Chenggong District, Kunming, Yunnan, China

**Keywords:** network pharmacology, non-small cell lung cancer, Pei Yuan Yi Fei decoction, prognostic model, RT-qPCR

## Abstract

**Objective:**

Although Pei Yuan Yi Fei Decoction (PYYFD) has been shown to be effective in treating NSCLC, the underlying mechanisms are not fully understood. This study aims to explore the possible molecular mechanisms underlying the therapeutic effects of PYYFD in NSCLC.

**Methods:**

Active compounds and their target genes were obtained from the TCMSP, GeneCards, and OMIM databases, and the intersecting genes of these genes were identified using a Venn diagram. Differentially expressed genes (DEGs) were identified from the transcriptomic dataset. Subsequently, the intersecting genes were intersected with the DEGs to identify candidate therapeutic targets. Based on the candidate therapeutic targets, univariate Cox regression and LASSO-Cox analyses were performed to evaluate their prognostic value. A prognostic risk model and nomogram were constructed and externally validated in GSE31210 and GSE42127. Single-cell RNA sequencing (scRNA-seq) was used to characterize the cell-type-specific expression patterns of these prognostic genes. Molecular docking was performed to assess potential compound-target interactions. RT-qPCR and western blotting were used to assess prognostic gene expression in PYYFD-treated PC-9 cells, while CCK-8 assays evaluated cell viability.

**Results:**

We identified 95 candidate therapeutic targets. LASSO analysis identified eight prognostic genes: CDC25C, GSTM2, BIRC5, EPHB2, NUF2, COL1A1, ADH1C and ACHE. This prognostic model demonstrated certain predictive capability in both internal and external cohorts (AUC > 0.6). scRNA-seq analysis showed that COL1A1 was specifically and highly expressed in fibroblasts. Molecular docking analysis suggested a computationally predicted potential interaction between COL1A1 and quercetin, with a predicted binding energy of approximately -9 kcal/mol. RT-qPCR and WB results showed that, after PYYFD treatment, the expression levels of all prognostic genes were significantly changed. CCK-8 results showed that PYYFD significantly reduced the viability of PC-9 cells.

**Conclusions:**

A total of 95 candidate therapeutic targets were identified in this study. Furthermore, eight prognostic genes (CDC25C, GSTM2, BIRC5, EPHB2, NUF2, COL1A1, ADH1C, and ACHE) were identified from the candidate therapeutic targets.

## 1 Introduction

Lung cancer (LC) continues to impose a substantial global cancer burden. It accounts for 12.4% of newly diagnosed malignancies and 18.7% of cancer-related deaths worldwide (1). According to the histopathological classification, LC is broadly categorized into small cell and non-small cell subtypes, with non-small cell lung cancer (NSCLC) comprising approximately 85% of all LC cases (2). NSCLC is usually diagnosed as lung adenocarcinoma or squamous cell carcinoma, characterized by high recurrence and metastasis rates (3). Furthermore, the poor prognosis and quality of life for NSCLC patients, who have a 5-year survival rate of only 15%, is the result of delayed diagnosis and limited treatment options (4). NSCLC is treated using the following current therapeutic strategies: surgery, chemotherapy, radiotherapy and immunotherapy (5). Significant progress has been made in targeted therapies, such as EGFR inhibitors, and immunotherapies, such as PD-L1 inhibitors, which have substantially improved patient survival and efficacy (6, 7), less than one-quarter of patients respond to these treatments (8). Also, problems with toxicity and drug resistance mean that they cannot be used very widely. Therefore, the urgent need for novel drugs with low toxicity for treating NSCLC is clear.

A treasure trove of natural remedies for thousands of years, Traditional Chinese Medicine (TCM) is a valuable source for the development of antitumor drugs. There is accumulating evidence that TCM has several advantages, including low toxicity, multiple therapeutic targets, a good safety profile, and significant efficacy (9). It has gained wide acceptance as a complementary or alternative therapy. PYYFD is a new formulation based on modified versions of the Jin Shui Liu Jun and Huang Qi Gui Zhi Wu Wu decoctions. Based on the TCM theory of eliminating pathogens and strengthening vital qi, PYYFD is combined according to the therapeutic strategies of Qi tonification and blood activation. Comprising 13 herbs including Danggui (Angelica sinensis), Huangqi (Astragali radix), Chenpi (Citri reticulatae pericarpium), and Banxia (Pinellia ternata), PYYFD’s anticancer effects have been validated through years of clinical practice. Experimental studies have confirmed that components of Angelica sinensis (Z-ligustilide and ligustilide) have anti-cancer properties, which they exert by regulating the cell cycle of NSCLC cells and promoting apoptosis (10, 11). The effective elimination of NSCLC cells (A549 cells) is achieved by Citri Reticulatae Pericarpium, without any toxicity to normal lung fibroblasts (MRC-5 cells) (12). Fan et al. demonstrated, through a series of cellular experiments, that Pinellia ternata significantly inhibits the proliferation, migration and invasion of NSCLC cells (13). Astragalus membranaceus is a plant commonly used in anticancer therapy and has been shown to have potent anticancer effects against various cancers, including NSCLC (14). Furthermore, it enhances chemotherapy efficacy by boosting systemic immunity and modulates the tumor immune microenvironment to alleviate immunosuppression (15). Previous studies have demonstrated that these individual herbs in PYYFD exert anti-NSCLC effects to varying degrees. However, the overall mechanism of action of PYYFD in NSCLC remains insufficiently investigated.

Network pharmacology, as an interdisciplinary research approach integrating pharmacology, systems biology, and bioinformatics, effectively incorporates the fundamental principles of TCM and provides a reliable tool for systematically elucidating the complex mechanisms underlying the therapeutic effects of Chinese herbal medicine (16). In addition, network pharmacology has brought about a paradigm shift in TCM research, moving from the traditional and simplified “single target-drug” model to a systematic and comprehensive “compound-target-disease” framework (17). Simultaneously, with the rapid development of high-throughput sequencing technologies, databases including TCGA, GEO, and GTEx offer powerful resources for systematically studying gene expression profiles and clinical characteristics in large sample cohorts (18). Therefore, an increasing number of researchers have applied network pharmacology combined with bioinformatics to investigate the anti-tumor mechanisms of TCM. For example, Xiao et al. found through network pharmacology analysis that Astragali Radix may exert anti-NSCLC effects through pathways such as JAK-STAT and PI3K-AKT (14). Another study showed that Yiwei decoction may exert therapeutic effects against NSCLC by targeting CCND1, EGFR, and TP53, thereby affecting stress-related signaling pathways such as HIF-1α (19). Although these studies have partly clarified the molecular mechanisms underlying TCM effects, experimental validation and multi-omics evidence remain insufficient. Meanwhile, few studies have further explored the effects of TCM on the complex tumor microenvironment (TME) of NSCLC. NSCLC is characterized by a complex TME and cellular heterogeneity, and the TME is also a major factor determining therapeutic response and clinical outcomes (20). scRNA-seq technology provides a foundation for investigating the complex TME (21). The scRNA-seq is increasingly being applied in TCM research to reveal the coordinated regulation of cellular networks by TCM (22). A recent study found that quercetin, a key component of Yu Ping Feng San, may treat NSCLC by targeting AHSA1 and modulating macrophage polarization (23).

Therefore, this study integrates network pharmacology, bioinformatics, scRNA-seq, molecular docking, and experimental validation to explore the active compounds, candidate therapeutic targets, and potential mechanisms underlying the anti-NSCLC effects of PYYFD. In addition, we explored the prognostic value of these candidate therapeutic targets. This study aims to provide a reference for the treatment of NSCLC with PYYFD.

## 2 Methods

### 2.1 Data acquisition and preprocessing

From the UCSC Xena platform (https://xena.ucsc.edu/), RNA-seq data, clinical information, and survival data of lung squamous cell carcinoma (LUSC) and lung adenocarcinoma (LUAD) were retrieved. LUSC and LUAD datasets were merged into a combined cohort designated TCGA-NSCLC (training set), comprising 1,019 tumor samples and 110 adjacent normal tissue samples. Tumor samples were filtered to retain only those with: Complete survival time >0 days, documented survival status, removal of duplicate patient entries. In addition, samples with missing data were excluded. Following preprocessing, 990 tumor samples were included for subsequent analysis. The datasets GSE198099, GSE31210, and GSE42127 were obtained from the GEO database, with GSE31210 (226 tumor samples) and GSE42127 (176 tumor samples) serving as independent validation sets. The baseline clinical characteristics of the training and validation cohorts are presented in **Supplementary Tables 1**–**3**. The NSCLC scRNA-seq dataset GSE198099 included two tumor and two paired normal tissue samples.

The TCMSP database (https://tcmsp-e.com/tcmsp.php) was used to obtain the active compounds and target genes of PYYFD, with the following screening criteria: oral bioavailability (OB) ≥ 30%, drug-likeness (DL) ≥ 0.18, and half-life (HL) ≥ 4. NSCLC-associated disease targets were retrieved from GeneCards (https://www.genecards.org/) and OMIM (https://omim.org/), and filtered by retaining protein-coding genes with a relevance score > 1. After integration and deduplication, these genes were used as the final target gene set for subsequent analysis.

### 2.2 Identification of candidate therapeutic targets

Differentially Expressed Genes (DEGs) were identified in R using DESeq2 (v1.44.0), applying significance thresholds of |log2 FC| ≥ 1 and adj.p< 0.05. The intersection of DEGs, Drug-target genes, as well as NSCLC disease targets were defined as candidate therapeutic targets.

### 2.3 Functional enrichment analysis

The clusterProfiler R package (v4.12.0) was employed for GO and KEGG pathway enrichment analysis of candidate therapeutic targets. Terms showing a statistically significant enrichment (adj.p< 0.05) were identified to reveal associated biological functions and signaling pathways of the candidate therapeutic targets. In addition, a PPI network was constructed using the STRING database (https://cn.string-db.org/), with a confidence score > 0.4 and the species set as “Homo sapiens”.

### 2.4 Prognostic model construction

The TCGA-NSCLC cohort served as the training set. Candidate therapeutic targets were included in univariate Cox regression analysis employing the “survival” R package (v3.5-7) to get survival-associated genes (HR ≠ 1, p< 0.05). Subsequently, the “glmnet” package (v4.1-8) was used to further screen the above genes to identify prognostic genes. To ensure the accuracy and stability of the model, 10-fold cross-validation was performed. Meanwhile, the optimal λ value was selected based on the minimum criterion to identify more representative genes. Sample-specific risk scores were computed as:


$$\text{Riskscore }=\;\sum \text{coef }\left(\text{genei}\right)\;*\text{ expr }\left(\text{genei}\right)$$

Tumour samples were stratified into high- and low-risk groups using median risk scores. Prognostic discrimination assessed through Kaplan-Meier survival curves and time-dependent receiver operating characteristic (ROC) analysis. To further evaluate the model’s robustness and generalizability, external validation was performed in GSE31210 and GSE42127 cohorts.

### 2.5 Gene set enrichment analysis (GSEA)

DEGs between risk groups were identified using DESeq2 (v1.44.0) with the criteria of |log2FC| ≥ 1 and FDR< 0.05. Raw P-values were adjusted for multiple comparisons using the Benjamini-Hochberg false discovery rate procedure. GSEA was conducted using “clusterProfiler” (v4.12.0) to obtain significantly enriched pathways between risk groups (adj.p< 0.05).

### 2.6 Single-Cell RNA sequencing quality control

GSE198099 data analysis were performed using the Seurat (v5.2.1) in R. Low quality cells were then excluded from the GSE198099 data according to the following quality control criteria: min.cells≥10 cells, 100< nCount_RNA)< 2000, percentage.mt≥10%. Next, the NormalizeData function was used for data normalisation, and FindVariableFeatures function identifies highly variable genes (0.1< mean expression< 3, dispersion > 0.5). Batch correction employed Harmony (v1.2.3) in R to eliminate batch effects from interfering with downstream analysis.

### 2.7 Cell clustering and annotation

Unsupervised clustering was conducted employing FindNeighbors and FindClusters (resolution: 0.1-1.5) function. Uniform Manifold Approximation and Projection (UMAP) visualized clusters. Marker genes were identified (FindAllMarkers, log2FC >0.25, adj. p<0.05) and cell types annotated using canonical markers (24).

### 2.8 scRNA-seq analysis

Cell type proportions in tumor versus adjacent normal tissues were compared via Wilcoxon rank-sum test. Cell types showing significant abundance differences (adj. p<0.05) were identified. Expression patterns of prognostic genes within these cell types were analyzed. Subsequently, prognostic genes highly expressed in differential cell populations were subjected to the Wilcoxon rank-sum test to determine the differences in expression levels between the tumor and normal groups.

In addition, to further investigate the immune microenvironment in NSCLC, we conducted cell-cell communication and pseudotime analysis. CellChat (v1.6.1) inferred intercellular signaling networks. Pseudotime trajectories were inferred employing Monocle2 (v2.30.0).

### 2.9 Molecular docking

Genes exhibiting consistent expression patterns between single-cell analysis and transcriptomic analysis were selected for molecular docking analysis. Meanwhile, the active compounds with the highest number of targets were selected. The three-dimensional structures of gene encoded proteins were retrieved from the PDB database (https://www.rcsb.org/). Protein crystal structures underwent hydrogen bonding optimization using PyMOL software and were converted to PDBQT format for use as molecular docking receptors. The two-dimensional structure of active components was downloaded from PubChem (https://pubchem.ncbi.nlm.nih.gov/), then formatted as a PDBQT ligand. AutoDock Vina within AutoDock Tools (v1.5.6) was used to perform molecular docking, which yielded the binding affinity between protein receptors and ligands (25). The detailed molecular docking parameters are presented in **Supplementary Table 4**.

### 2.10 Preparation of PYYFD solution and cell culture

PYYFD was composed of Angelica Sinensis Radix, prepared Rehmannia root, Pericarpium Citri Reticulatae, Arum Ternatum Thunb, Poria, raw Radix Astragali, Adenophorae Radix, Fritillariae Thunbergii Bulbus, Ramulus Cinnamomi, Paeoniae Radix Alba, Spina Gleditsiae, Hedyotis diffusa, and Radix Glycyrrhizae Preparata, with a total weight of 171 g, and was prepared as granules. The granule formulation was produced by Guangdong Yifang Pharmaceutical Co., Ltd. (batch number: 2602001W). For preparation of PYYFD solution, 10 g of granules were accurately weighed and dissolved in 100 mL of distilled water (1 g:10 mL). The mixture was thoroughly stirred until complete dissolution and subsequently concentrated to a final volume of 50 mL under reduced pressure. The resulting PYYFD stock solution was therefore obtained at a concentration of 200 mg/mL. The solution was sterilized using a 0.22 μm membrane filter and stored at 4 °C until further use. Before the formal experiments, CCK-8 time–concentration gradient experiments were performed at 24, 48, and 72 h, with a water vehicle control and a blank control group included (n = 3 per group). The results are presented in **Supplementary Table 5** and **Supplementary Figure 1**. Based on the preliminary experimental results, 1, 8, and 16 mg/mL were selected as the low, medium, and high doses, respectively. For cell experiments, the stock solution was diluted with RPMI-1640 medium to obtain final concentrations of 1, 8, and 16 mg/mL.

The human NSCLC cell line PC-9 (ZQ0487, Shanghai, China) was obtained from Shanghai Zhong Qiao Xin Zhou Biotechnology Co., Ltd. (Shanghai, China). Cells were cultured in RPMI 1640 (RPMI-1640, Daling) medium supplemented with 10% fetal bovine serum (FBS) (DLH401-500, Daling) and 1% penicillin-streptomycin (0513, Daling). Cells were incubated at 37 °C in a humidified atmosphere containing 5% CO_2_.

### 2.11 CCK-8

The effect of PYYFD on PC-9 cell viability was assessed using the Cell Counting Kit-8 (CCK-8) assay. Cells were exposed to culture medium containing different concentrations of PYYFD for 0, 24, 48, and 72 h. Subsequently, 10 μL of CCK-8 solution (BS350A, Biosharp) was added to each well, followed by incubation for 2 h. The optical density (OD) at 450 nm was measured using a microplate reader (Thermo). All experiments were independently repeated at least three times.

### 2.12 RT-qPCR and western blot

Genes highly expressed in cell populations exhibiting significant abundance alterations in the scRNA-seq analysis were further validated by Western blotting. Total protein was extracted using RIPA lysis buffer (proteintech, Cat No. PR20035) from PC-9 cell. Protein concentration was determined using a BCA Protein Assay Kit (Proteintech, Cat No. PK10026), and then Western blotting was performed. The antibodies used for western blotting included rabbit anti-NUF2 (bsm-61893R, Bioss, China), rabbit anti-BIRC5 (bsm-54475R, Bioss, China), rabbit anti-COL1A1 (bs-10423R, Bioss, China), and rabbit anti-GAPDH (bsm-52262R, Bioss, China) and Goat anti-Rabbit IgG (bs-0295G, Bioss, China). The mean gray value of Western blot images was measured using ImageJ/Fiji software. Each band was measured three times, and the mean value was calculated. The relative expression level of the target protein = mean gray value of the target protein/mean gray value of the internal GAPDH. Each experimental group included three independent biological replicates (n = 3), and the results were presented as mean ± standard deviation. All bands were measured under the same exposure conditions and identical analysis parameters.

Total RNA was extracted using TRNzol Universal (TIANGEN, Cat No. DP404). First-strand cDNA synthesis and qPCR were performed. The primer sequences are listed in **Supplementary Table 6**. GAPDH was used as the internal reference gene. Relative gene expression was calculated using the 2^^−ΔΔCt^ method.

### 2.13 Statistical analysis

R software (version 4.4.1, https://www.r-project.org/) was utilized for all statistical analyses. For comparisons between two groups, Student’s t-test was used for normally distributed data, whereas the Wilcoxon rank-sum test was used for non-parametric data. For comparisons involving multiple groups, one-way analysis of variance (ANOVA) was performed. Overall survival was assessed using Kaplan-Meier curves and the log-rank test. Univariate and multivariate Cox proportional hazards regression analyses were used to calculate hazard ratios and 95% confidence intervals, while LASSO-Cox regression was performed using the glmnet package to select prognostic genes. For differential expression, enrichment, GSEA, and scRNA-seq analyses, multiple comparisons were controlled using adj.p. Quantitative data from at least three independent experiments are presented as the mean ± standard deviation. Statistical Significance was established at P< 0.05. *, **, and *** denoting p<0.05, p<0.01, and p<0.001, respectively.

## 3 Results

### 3.1 Identification of differential gene expression

Differential analysis of NSCLC samples from UCSC Xena revealed 6394 DEGs, with 4016 upregulated and 2378 downregulated in tumor tissues (|log2 FC| ≥ 1, adj.p< 0.05) (**Figure 1**).

**Figure 1:**
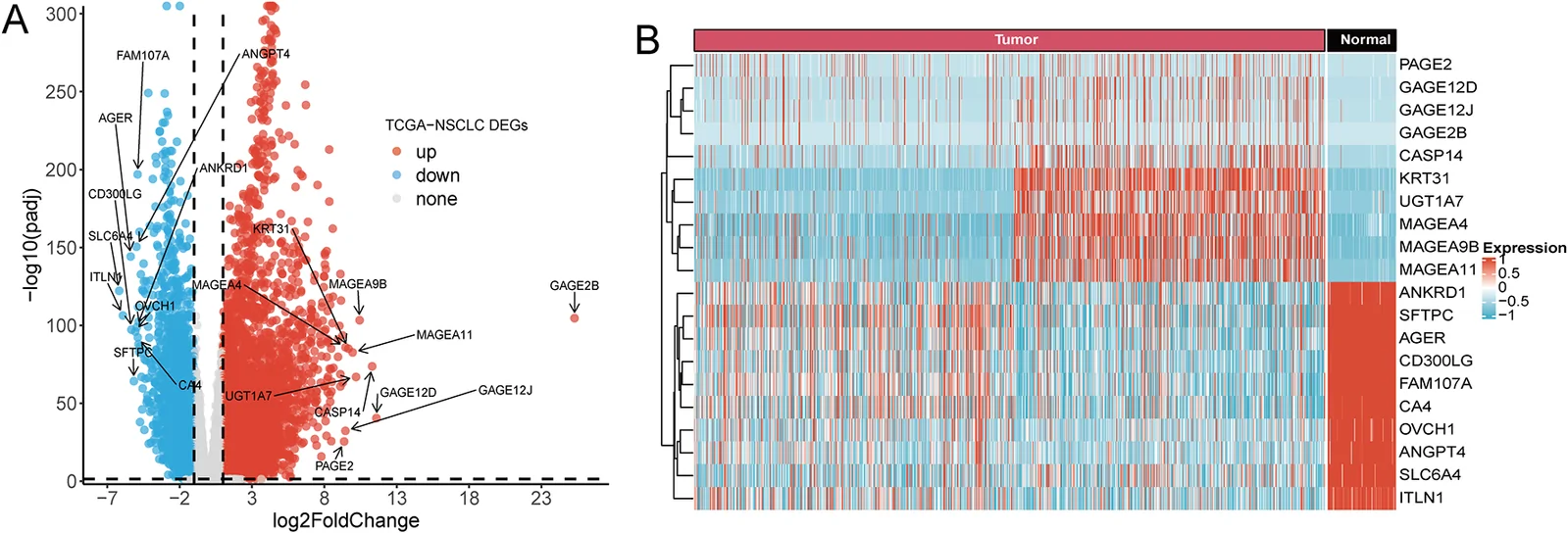
TCGA-NSCLC differential genes. **(A)** Volcano plot of differentially expressed genes; **(B)** heatmap of top 10 up- and down-regulated DEGs.

### 3.2 Construction of the herb-ingredient-target network

From TCMSP, 111 active drug ingredients (OB ≥ 30%, DL ≥ 0.18, and HL ≥ 4) and 256 corresponding targets were identified. Totaling 5912 disease targets was obtained, included 5772 from GeneCards as well as 192 from OMIM. Intersection analysis yielded 203 intersecting genes (**Figure 2A**). A Herb-Ingredient (MOL)-Target network was constructed (**Figure 2B**), comprising 13 herbs, 111 ingredients, 203 intersecting genes, and 1589 interaction pairs.

**Figure 2:**
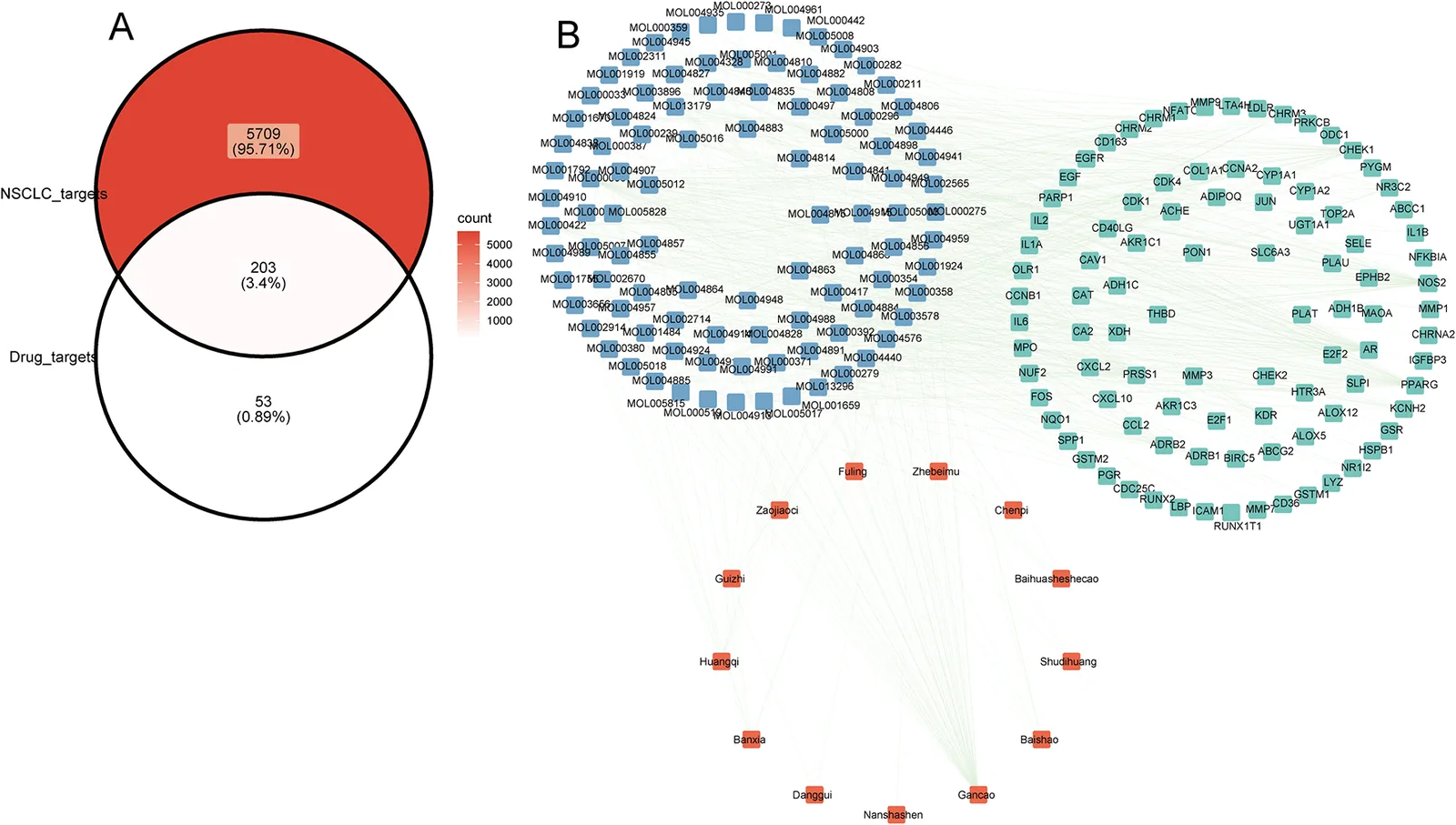
Prediction intersecting genes of PYYFD for NSCLC treatment. **(A)** Venn diagram of intersecting genes; **(B)** Herb - active ingredient - targets network.

### 3.3 Identification and functional analysis of candidate therapeutic targets

A total of 95 candidate therapeutic targets were identified after intersecting genes were intersected with DEGs (**Figure 3A**; **Supplementary Table 7**). GO enrichment analysis demonstrated significant involvement in stress/immune responses, including lipopolysaccharide (LPS), bacterial molecules, xenobiotics, oxidative stress, nutrients, and chemical stimuli (**Figure 3B**). KEGG analysis revealed enrichment in TNF, IL-17, NF-κB, Toll-like receptor, and cellular senescence pathways (**Figure 3C**). The STRING dataset (confidence: 0.4) was utilized to construct the PPI network of candidate therapeutic targets, which contained 95 nodes and 859 interaction pairs (**Figure 3D**).

**Figure 3:**
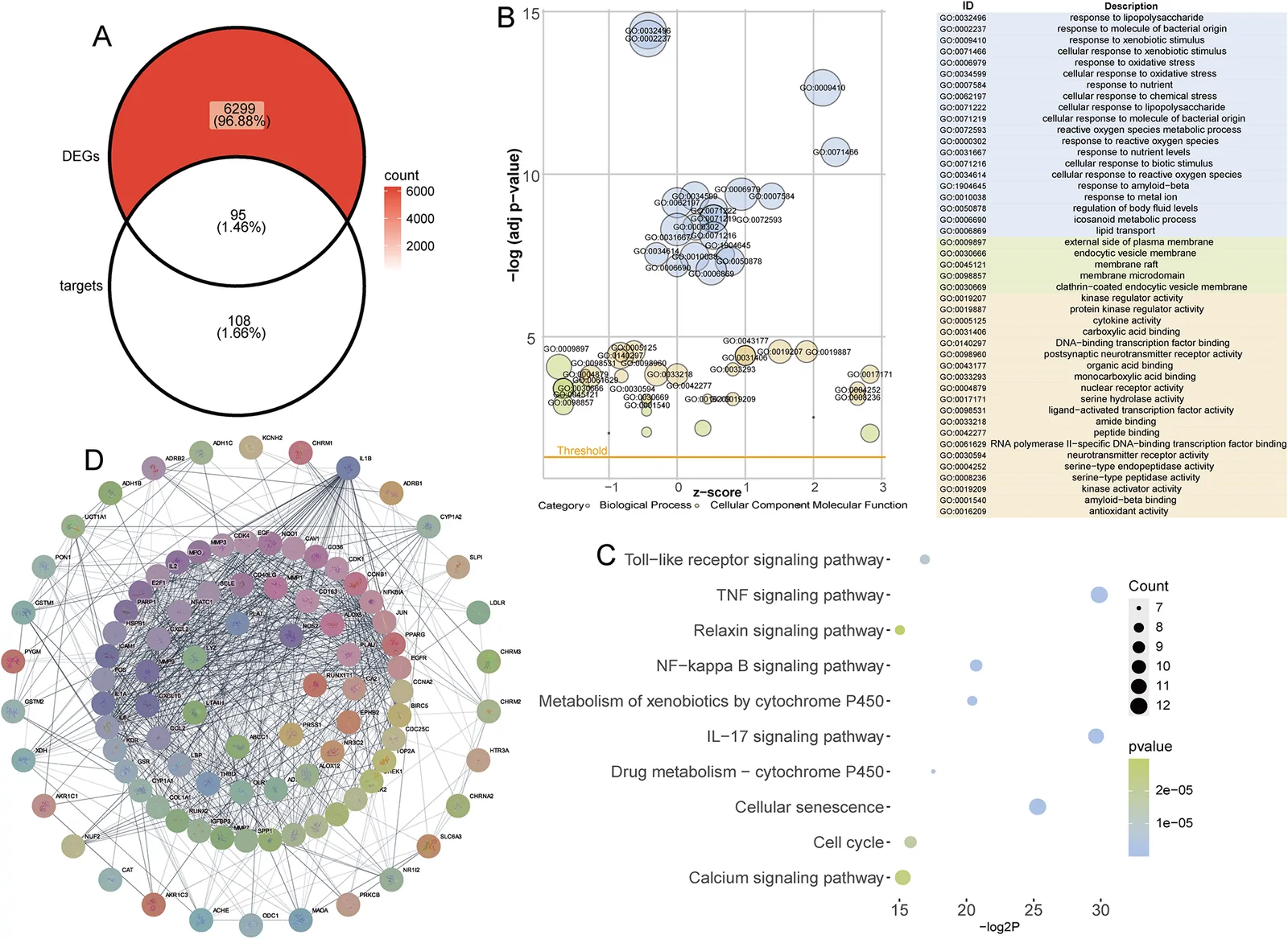
Functional annotation and identification of candidate therapeutic targets. **(A)** Venn diagram of candidate therapeutic targets; **(B)** GO functional enrichment bubble plot of candidate therapeutic targets; **(C)** Sankey diagram of KEGG pathways enriched by candidate therapeutic targets; **(D)** PPI network of candidate therapeutic targets.

### 3.4 Prognostic model construction and validation

Using TCGA-NSCLC as the training set, GSE31210 and GSE42127 as validation sets, we further analyzed the prognostic characteristics of candidate therapeutic targets. Univariate Cox regression of 95 candidate therapeutic targets identified 22 survival-associated genes (P< 0.05) (**Figure 4A**). Seventeen genes meeting the PH assumption underwent LASSO-Cox regression, retaining eight prognostic genes with non-zero coefficients, including CDC25C, GSTM2, BIRC5, EPHB2, NUF2, COL1A1, ADH1C, and ACHE (**Figure 4B**). The univariate Cox regression results for the prognostic genes are shown in **Supplementary Table 8**. The detailed results of the LASSO analysis are presented in **Supplementary Table 9**. An important consideration is that performing both model training and feature selection in the same TCGA-NSCLC cohort may lead to optimism bias. Subsequently, the risk score was calculated according to the following formula: Riskscore = 0.096*CDC25C + (-0.074)*GSTM2 + 0.050*BIRC5 + 0.107*EPHB2 + 0.010*NUF2 + 0.035*COL1A1 + (-0.026)*ADH1C + (-0.039)*ACHE.

**Figure 4:**
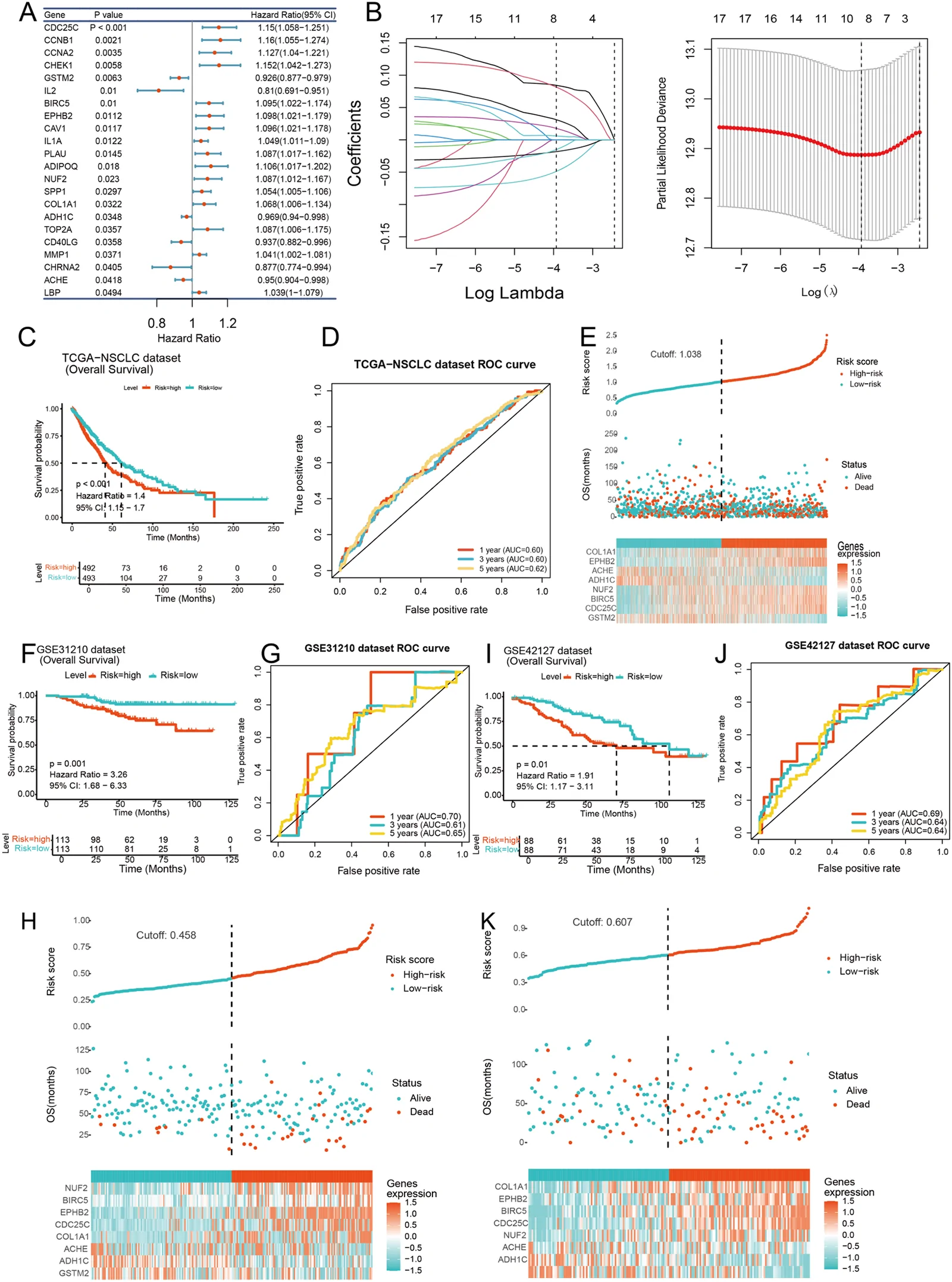
Construction and validation of the NSCLC prognostic model. **(A)** Univariate Cox regression forest plot demonstrating 22 survival-related genes; **(B)** LASSO coefficient trajectories (left) and cross-validation error (right) for feature selection; **(C)**: Kaplan-Meier survival curves stratified by risk score (high-risk: red; low-risk: blue); **(D)** Time-dependent ROC curves assessing model accuracy (TCGA-NSCLC cohort); **(E)** Risk stratification plots: survival curves (top), survival status (middle), and signature gene expression heatmap (bottom) (TCGA-NSCLC cohort); **(F)** Kaplan-Meier survival analysis (GSE31210); **(G)** ROC curve (GSE31210); **(H)** Risk stratification plots (GSE31210); **(I–K)** External validation using GSE42127. **(I)** Survival curves; J, ROC analysis; K, Risk stratification.

K-M analysis revealed significantly worse prognosis in high-risk patients (HR = 1.40, 95% CI = 1.20-1.63, P< 0.001), **Figure 4C**. As shown in **Figure 4D**, the 1-, 3-, and 5-year AUC values were 0.60, 0.60, and 0.62, respectively. In addition, the scatter plot showed a higher mortality rate among patients in the high-risk group (**Figure 4E**). Validation in GSE31210 and GSE42127 yielded consistent results(**Figures 4F–K**). Specifically, in the GSE31210 dataset, patients in the high-risk group had poorer OS (HR = 3.26, 95% CI = 1.68-6.33, P = 0.001), with 1-, 3-, and 5-year AUC values of 0.70, 0.61, and 0.65, respectively. In the GSE42127 dataset, patients in the low-risk group had better OS (HR = 1.91, 95% CI = 1.17-3.11, P = 0.01), with corresponding 1-, 3-, and 5-year AUC values of 0.69, 0.64, and 0.64, respectively.

### 3.5 Independent prognostic analysis and nomogram validation

To evaluate the risk score (based on 8 prognostic genes) as an independent prognostic factor, we performed Cox PH tests incorporating clinical variables (age, gender, TNM stage, clinical stage). Risk score, age, TNM stage, and clinical stage passed PH assumptions (HR > 1), **Figure 5A**. Subsequent multivariate Cox regression confirmed risk score (HR = 1.8, 95% CI = 1.34 - 2.4, P< 0.001) and age (P = 0.013) as independent risk factors, **Figure 5B**. A nomogram integrating age and risk score predicted survival probability (**Figure 5C**). Calibration curves showed that the 1- and 3-year nomogram-predicted probabilities were generally consistent with the observed outcomes (**Figure 5D**). The 1-, 3-, and 5-year AUC values of 0.609, 0.607, and 0.604, respectively (**Figure 5E**). These results indicate that the nomogram has a limited predictive capability.

**Figure 5:**
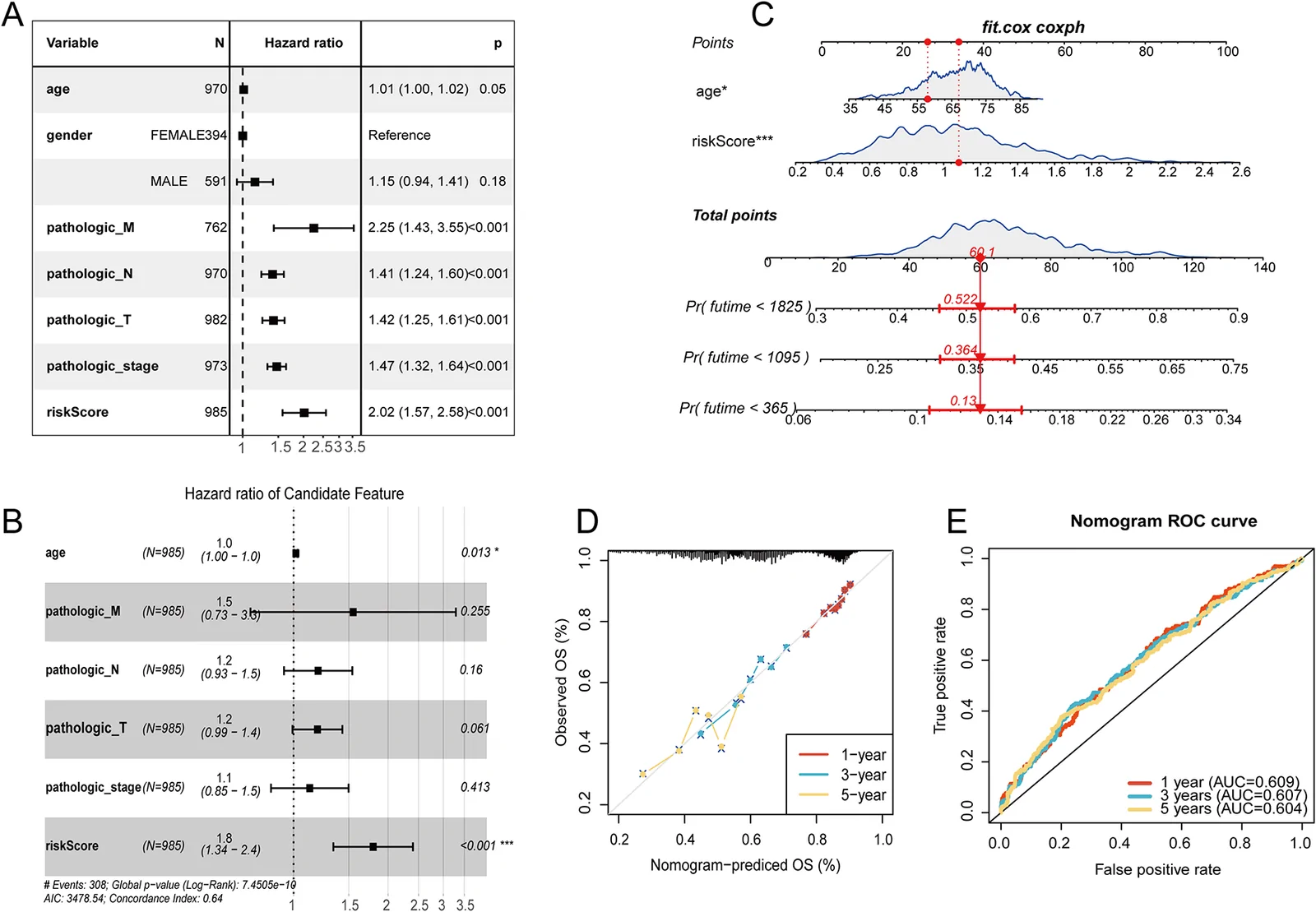
Independent prognostic value of the risk score. **(A)** Univariate Cox regression forest plot of clinical factors; **(B)** Multivariate Cox regression identifying independent predictors; **(C)** Nomogram integrating age and risk score for survival prediction; **(D)** Calibration curve comparing predicted vs. actual survival; **(E)** ROC curve.

### 3.6 GSEA functional enrichment of risk groups

GSEA of risk groups revealed distinct functional profiles. High-risk group enriched in cell cycle, Fanconi anemia pathway, ECM - receptor interaction, homologous recombination, DNA replication, and mismatch repair (**Figure 6A**). While low-risk group enriched in aldosterone - regulated sodium reabsorption, ascorbate and aldarate metabolism, carbohydrate digestion and absorption, as well as drug metabolism - cytochrome P450 (**Figure 6B**). These findings indicate that significant differences in biological function between two groups, and prognostic genes may drive NSCLC progression by modulating cell cycle dynamics and replicative pathways.

**Figure 6:**
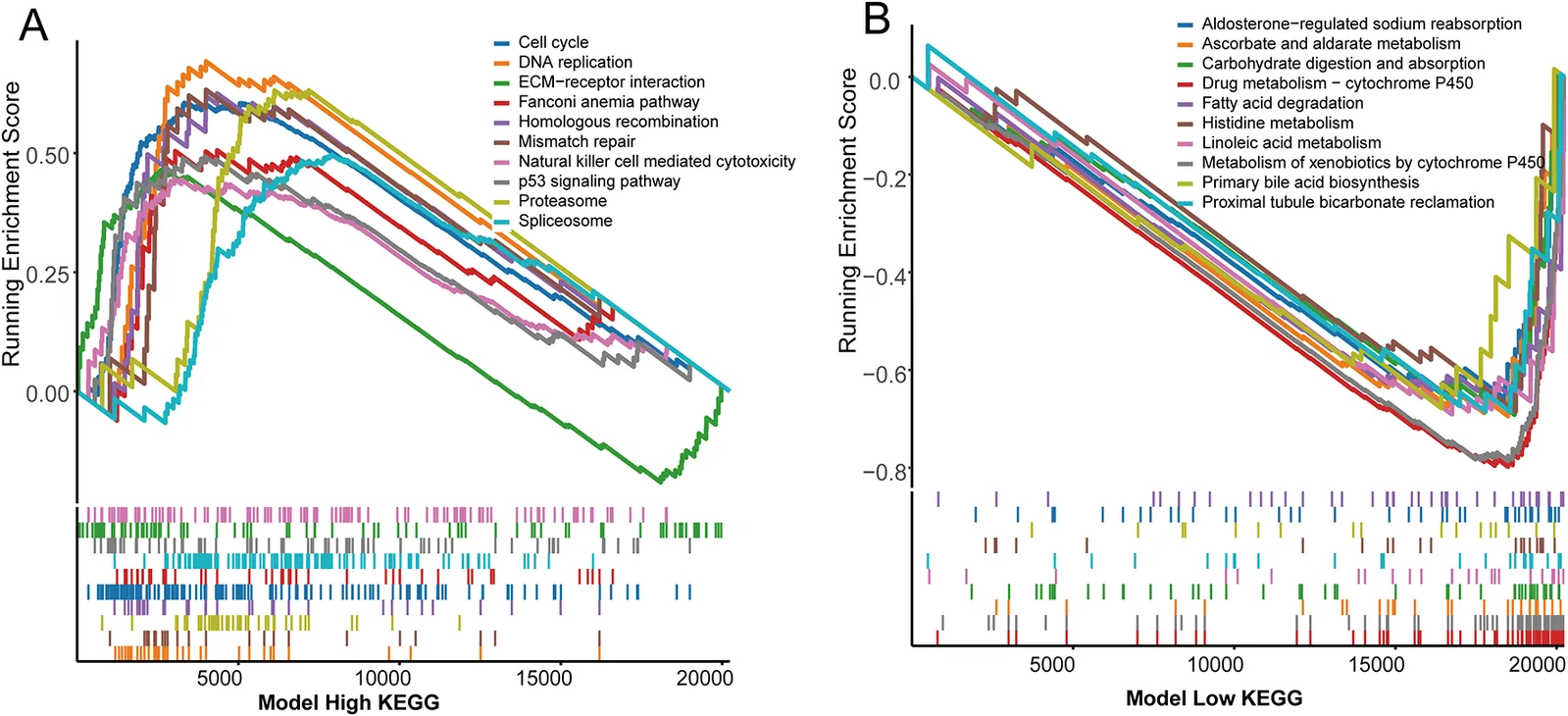
GSEA enrichment in risk subgroups. **(A)** Enriched pathways ridge plot in high-risk group; **(B)** Enriched pathways ridge plot in low-risk group.

### 3.7 scRNA-seq analysis

After quality control, single-cell RNA-seq data (GSE198099) comprised 21,244 genes across 20,848 cells (**Supplementary Figure 2A**). Highly variable genes were identified, with the top 10 labeled (**Supplementary Figure 2B**). PCA reduced dimensionality (**Supplementary Figure 2C**), retaining the top 30 principal components for clustering (**Supplementary Figure 2D**). All cells were clustered into 15 clusters by unsupervised cluster analysis (resolution 0.1), **Figure 7A**, and further annotated into ten cell types: T cells (CD2, CD3D, CD3E, CD3G, TRAC, CD8A), Tumor-associated fibroblasts (C1R, COL1A2, DCN), Monocytes (C1orf54, LGALS2, CD68, CD300E, CXCL8), Tumor cells (ELF3, KRT8, EPCAM, KRT18, KRT19), B cells (CD19, CD79A, MS4A1), NK cells (GNLY, NKG7), Mast cells (KIT, MS4A2, PTGS1, RGS13), Endothelial cells (CDH5, CLDN5, RAMP2), Plasma cells (IGKC, IGLC3, IGLC2), Plasmacytoid dendritic cells (IRF4, GZMB), **Figures 7B, C**. Quantification revealed expanded NK cells, T cells, and fibroblasts in tumors (**Figure 7D**). Among the eight prognostic genes, BIRC5 and NUF2 showed relatively high expression in NK cells, whereas COL1A1 was preferentially expressed in fibroblasts (**Figure 7E**). Wilcoxon testing showed significantly higher COL1A1 expression in fibroblasts from tumor tissues than in those from adjacent normal tissues (P< 0.05; **Figure 7F**). Fibroblasts were therefore selected as a cell population of interest for subsequent exploratory analyses.

**Figure 7:**
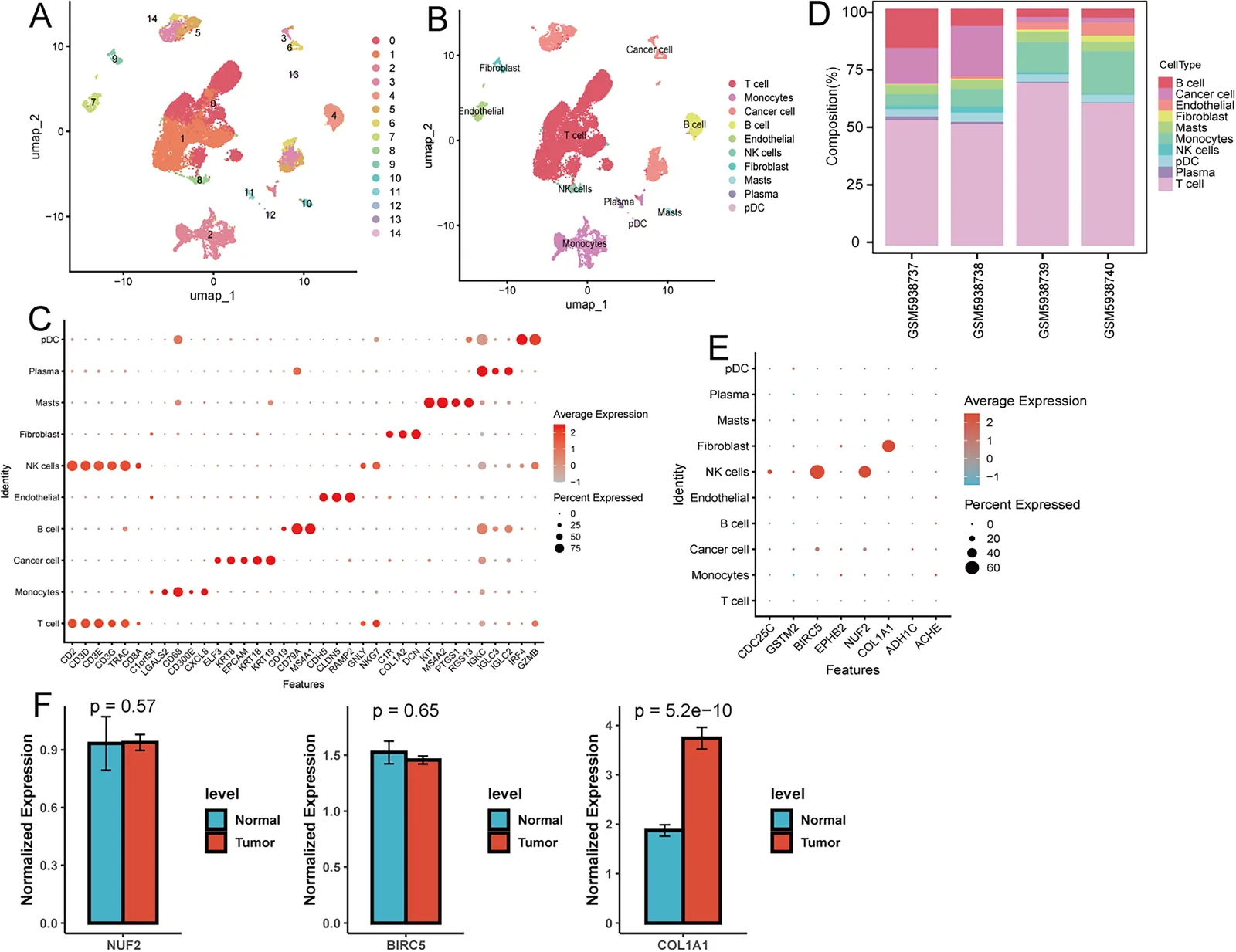
Single-cell analysis to obtain key cells. **(A)** UMAP visualization of 15 transcriptionally distinct cell clusters; **(B)** Annotation of 10 major cell subpopulations; **(C)** Dot plot displaying marker gene expression across 10 subpopulations; **(D)** Stacked bar plot comparing cell type proportions in tumor vs control samples (GSM5938737, GSM5938738, GSM5938739, GSM5938740); **(E)** Expression distribution of 8 prognostic genes across cellular subpopulations; **(F)** Differential expression of NUF2, BIRC5, and COL1A1 in tumor and normal tissues.

### 3.8 Fibroblast communication networks and trajectory analysis

CellChat analysis demonstrated robust fibroblast-tumor cell crosstalk (**Figure 8A**; **Supplementary Figure 3**). Fibroblast-specific signaling pathways (CD99, CXCL, THY1, SEMA4, COMPLEMENT, CD46, ANGPT) exhibited tumor-normal differential activity (**Figure 8B**). Fibroblasts is further dividedinto two subcluster: EMT-like (S100A4, MCAM, ACTA2), Myofibroblasts (COL5A1, COL1A1, COL1A2), **Figures 8C–E**. Then, Monocle2 pseudotime trajectory analysis showed Fibroblasts were divided into three different differentiation states, with State2 harboring the highest differentiation potential and EMT-like enrichment (**Figures 8F–H**). COL1A1 expression varied across the inferred trajectory and was relatively high in State 2 (**Figure 8I**).

**Figure 8:**
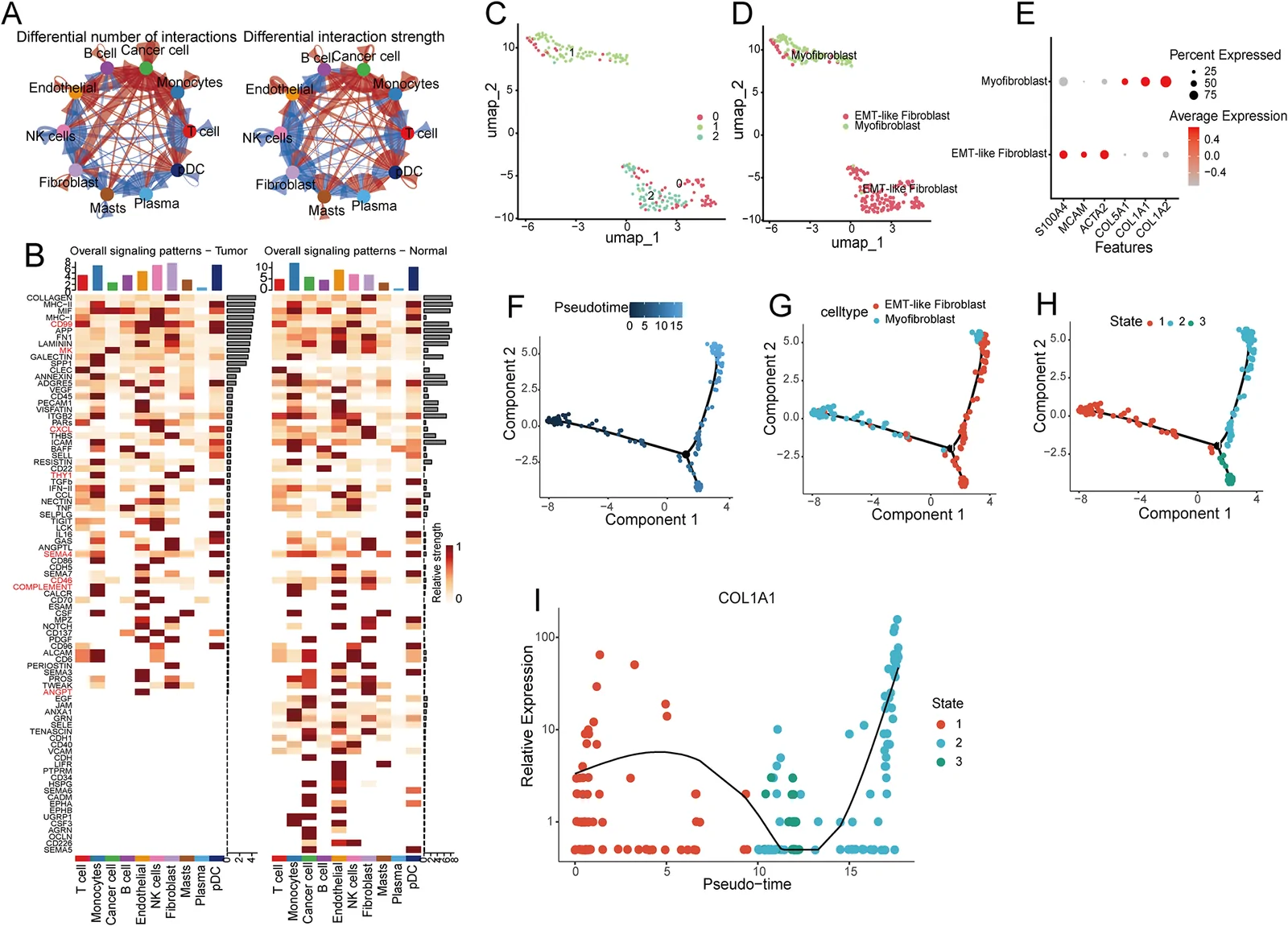
Cellular communication analysis and pseudotime analysis of Fibroblast. **(A)** Ligand-receptor interaction network between fibroblasts and other cells; **(B)** Heatmap of dysregulated signaling pathways in tumor-associated fibroblasts; **(C)** Three cell clusters were obtained after the first level classification; **(D)** Fibroblasts were annotated by cellular annotation into 2 cellular subpopulations: EMT-like fibroblasts (red) and myofibroblasts (blue); **(E)** Subtype-specific gene expression signatures. **(F)** Based on the pseudotime of Fibroblasts; **(G)** Cell distribution along pseudotime by subtype (red-orange color indicates EMT-like Fibroblast, blue indicates Myofibroblast); **(H)** The cell population is divided into three distinct states; **(I)** COL1A1 expression divergence across trajectory branches.

### 3.9 Molecular docking

We selected prognostic genes with consistent expression trends in the single-cell and transcriptomic analyses, together with active compounds targeting the largest number of targets, for molecular docking. Therefore, COL1A1 and quercetin were selected for molecular docking. The molecular docking information is presented in **Table 1**. Molecular docking results showed a computationally predicted potential interaction between quercetin and COL1A1, with a predicted binding affinity of −9.0 kcal/mol (**Figure 9**). The detailed molecular docking parameters are presented in **Supplementary Table 4**.

**Table 1:** Information of molecular docking analysis.

Herb_pinyin	MOL_ID	molecule_name	Target	ob	halflife	dl
Baihuasheshecao	MOL000098	quercetin	COL1A1	46.43	14.40	0.28
Huangqi	MOL000098	quercetin	COL1A1	46.43	14.40	0.28
Gancao	MOL000098	quercetin	COL1A1	46.43	14.40	0.28
Zaojiaoci	MOL000098	quercetin	COL1A1	46.43	14.40	0.28

**Figure 9:**
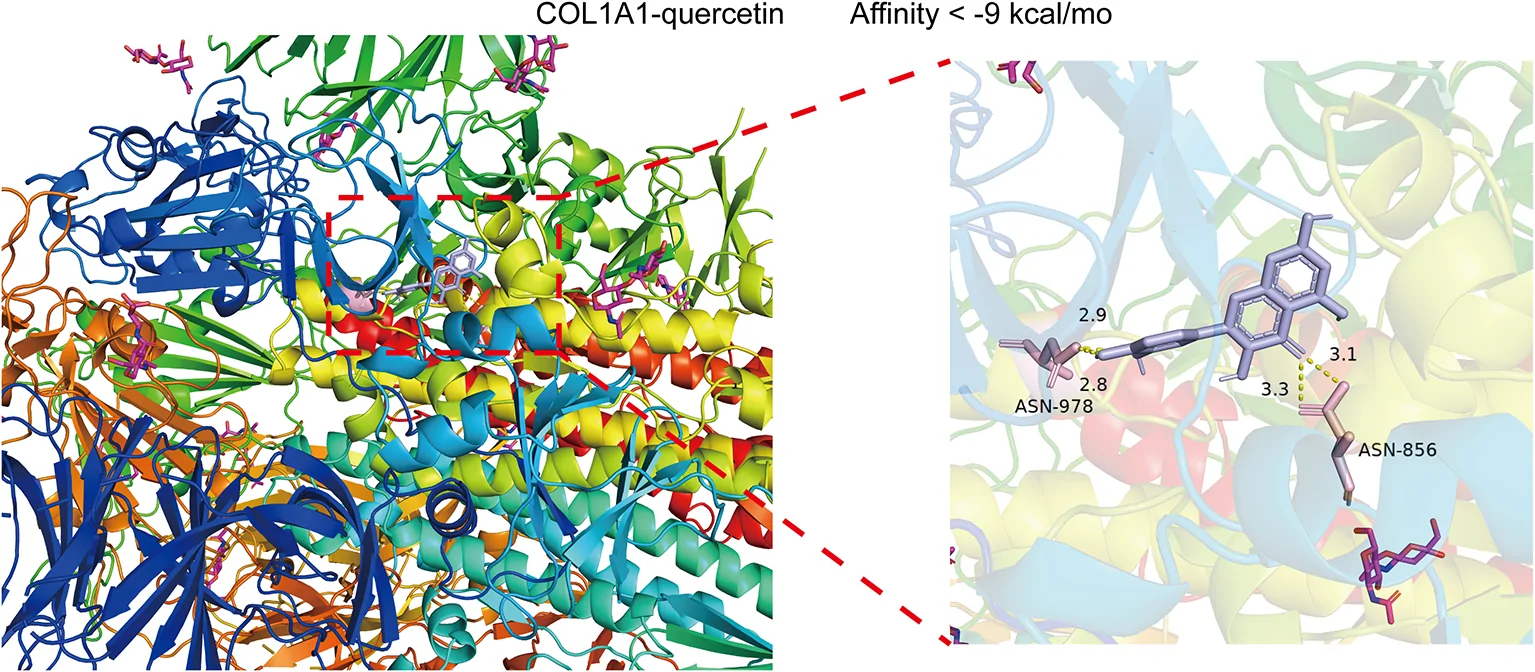
Molecular docking of quercetin with COL1A1.

### 3.10 Cell experiments

The preliminary experimental results are shown in **Supplementary Figure 1** and **Supplementary Table 5**. The vehicle control group showed no significant differences compared with the blank control group at 24, 48, or 72 h (P > 0.05), indicating that the water vehicle itself did not induce obvious cytotoxicity. At 24 h, treatment with 1 mg/mL PYYFD solution resulted in only slight inhibition of cell viability (86.91 ± 1.76%), whereas 8 mg/mL reduced cell viability to 66.08 ± 3.79%. After 72 h of treatment, cell viability further declined to 57.50 ± 2.22%. At 48 h, 1 mg/mL PYYFD solution did not exhibit apparent cytotoxicity (93.02 ± 0.53%), while 8 and 16 mg/mL PYYFD solution markedly reduced cell viability (51.46 ± 0.47% and 23.88 ± 0.96%, respectively). At 72 h, cell viability decreased in all treatment groups.

We used RT-qPCR to assess changes in prognostic gene expression after PYYFD treatment in PC-9 cells. The results showed that after treatment with different concentrations of PYYFD, with high, medium, and low concentrations set at 16 mg/mL, 8 mg/mL, and 1 mg/mL, respectively, the mRNA expression levels of ACHE, NUF2, EPHB2, BIRC5, and CDC25C were significantly decreased in a concentration-dependent manner, GSTM2 exhibited the opposite trend (P< 0.05, **Figures 10A–F**). In addition, compared with the control group, the expression levels of ADH1C and COL1A1 were also significantly reduced in the high-concentration group (P< 0.05, **Figures 10G, H**). Meanwhile, as shown in **Figures 10I–L**, after 48 h of PYYFD treatment, the protein expression levels of COL1A1, NUF2, and BIRC5 were decreased in the treatment group compared with the control group. Original Western blot images are shown in **Supplementary Figure 4**. The CCK-8 results showed that after treatment with different concentrations of PYYFD for 24, 48, and 72 h, the viability of PC-9 cells was inhibited, with the medium- and high-concentration groups showing the strongest effects and significant differences (P< 0.05, **Figure 10M**). Cellular morphological changes are illustrated in **Supplementary Figure 5**. However, these observations do not establish whether prognosis genes mediate the cellular response to PYYFD.

**Figure 10:**
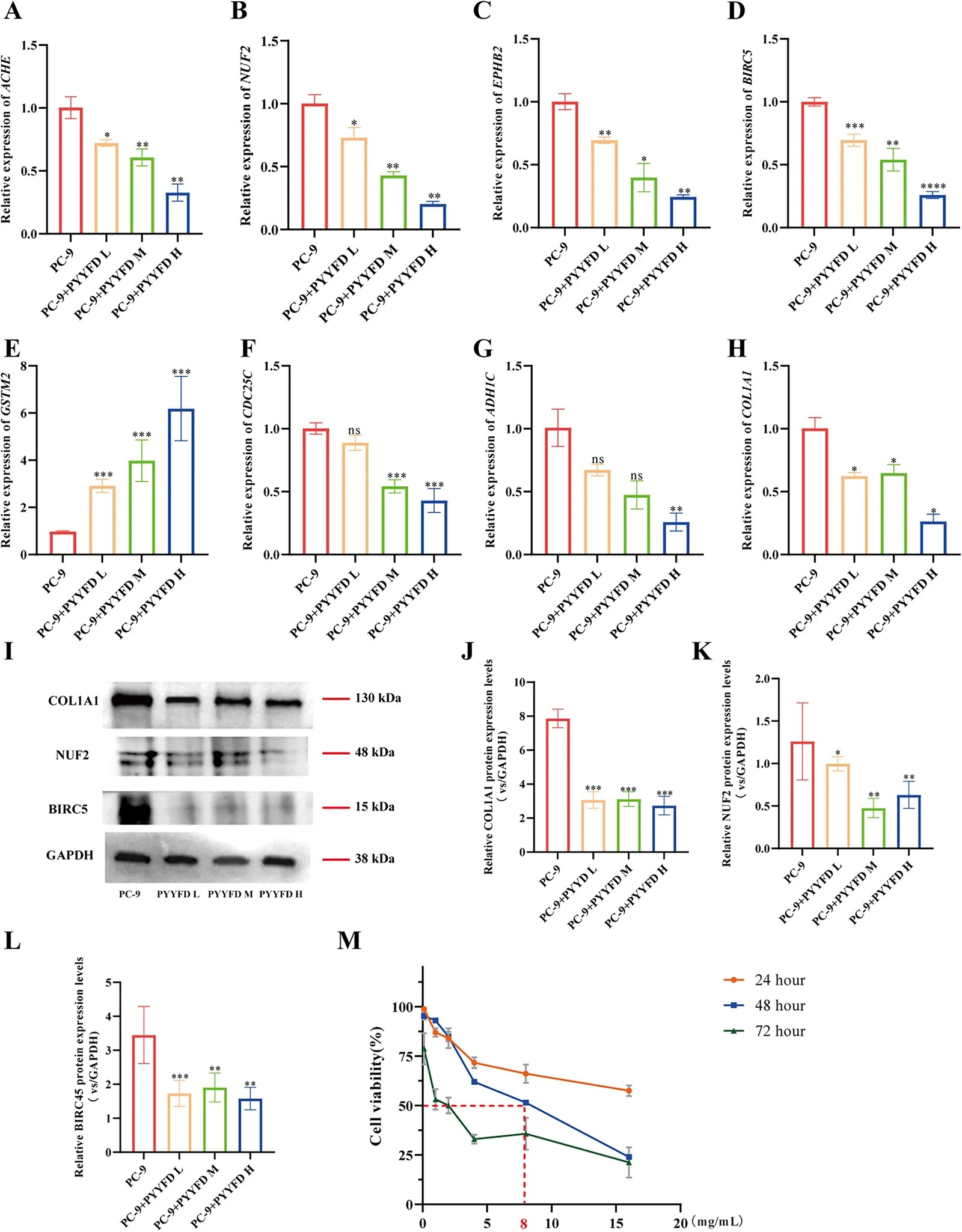
*In vitro* experimental based on PC-9 cells. **(A–H)** RT-qPCR results of prognostic genes, including ACHE, NUF2, EPHB2, BIRC5, GSTM2, CDC25C, ADH1C, and COL1A1, respectively. **(I)** WB image; **(J–L)** WB quantification; **(M)** CCK-8 assay. Three biological replicates were performed for the RT-qPCR and Western blot experiments.

## 4 Discussion

NSCLC is a disease with high recurrence, metastasis, and mortality rates. Due to its insidious early symptoms, patients are often diagnosed at advanced stages, resulting in delayed treatment opportunities and limited therapeutic options (26). Most patients can only be treated through chemotherapy, radiotherapy, targeted therapy, or immunotherapy. However, higher drug toxicity and adverse event rates lead to frequent dose adjustments and treatment discontinuation (27). In NSCLC treatment, TCM can effectively reduce treatment toxicity, enhance efficacy, alleviate symptoms, and improve patients’ quality of life (28). Nevertheless, as an effective therapeutic agent, PYYFD’s mechanisms in NSCLC remain unclear. Therefore, in this study, we explored the potential mechanisms underlying the therapeutic effects of PYYFD in NSCLC using network pharmacology and bioinformatics analyses. Finally, we preliminarily validated the analytical results at the cellular level.

In this study, we constructed an interaction network based on 203 therapeutic targets, 13 Chinese herbs, and their 111 active compounds. Of all the active components in this network, quercetin had the highest number of connected targets. Quercetin is a bioflavonoid, it is widely present in plants. Many studies demonstrate that it exerts anti-NSCLC effects by inducing cell death, inhibiting proliferation and migration, enhancing drug sensitivity and influencing drug resistance (29). Additionally, the top five active components by target connectivity were kaempferol, fisetin, baicalein, and naringenin. It has been confirmed by experimental results that NSCLC cell proliferation is significantly inhibited and autophagy is induced by kaempferol, potentially through the suppression of the PI3K/AKT signaling pathway (30, 31). Fisetin (3,3’,4’,7-tetrahydroxyflavone), which is a plant-derived flavonol, is really well known for its significant antitumour effects in various cancers, including NSCLC (32). Its antitumor activity primarily stems from antiproliferative and proapoptotic properties. Previous studies have demonstrated that fisetin exerts anti-NSCLC effects by upregulating CDKN1A/B and CDKN2D, thereby promoting cell-cycle arrest and apoptosis in NSCLC cells (33). A study using a NCI-H1299 xenograft nude mouse model discovered that baicalein causes MAP4K3 degradation, triggering TFEB nuclear translocation and autophagy activation, which results in the arrest of proliferation in LC cells (34). Naringenin, a citrus flavonoid, suppresses NSCLC cell viability and tumor growth through dual mechanisms of ROS-mediated apoptosis and autophagy, inhibiting CDK activity and activating the AMPK pathway (35).

GO and KEGG enrichment analyses revealed that the candidate targets were significantly enriched in biological processes related to cellular defense against external stimuli and stress responses. They were also found to be enriched in pathways involving inflammation, the immune response, the stress response, cell fate regulation and metabolism, including the TNF signaling pathway, cellular senescence and drug metabolism - cytochrome P450. The TNF signaling pathway plays crucial roles in key cellular processes including inflammatory response, immune regulation, and apoptosis (36, 37). In NSCLC, the TNF signaling pathway influences patient survival, tumor progression, and drug resistance (38, 39). Cellular senescence has dual effects: the persistent and irreversible cell cycle arrest it induces is considered an endogenous tumor suppression mechanism, while the senescence-associated secretory phenotype protects malignant cells from immune clearance, promoting cancer recurrence and metastasis (40). Drug metabolism - cytochrome P450 plays a vital role in TCM treatment of NSCLC, participating in regulating cell proliferation and apoptosis, oxidative stress, drug metabolism, and other processes (41). Therefore, these enrichment results reveal potential regulatory networks through which PYYFD treats NSCLC.

Subsequently, we further screened candidate therapeutic targets using LASSO and univariate Cox regression, ultimately identifying eight prognostic genes: CDC25C, GSTM2, BIRC5, EPHB2, NUF2, COL1A1, ADH1C, ACHE. Cell division cycle 25C (CDC25C) is a phosphatase critical for G2/M phase transition (42). Several studies have indicated that CDC25C is involved in regulating cell-cycle progression in NSCLC cells. An *in vitro* functional study demonstrated that CDC25C knockdown significantly reduced tumor cell proliferation, migration, and invasion and induced apoptosis (43). This is consistent with our *in vitro* findings, in which CDC25C expression and cell viability were significantly decreased after PYYFD treatment in PC-9 cells. In addition, CDC25C has also been reported as a therapeutic target of other drugs for NSCLC treatment. For example, *in vitro* and *in vivo* experiments by Xue et al. showed that Cyclovirobuxine D, a natural steroidal alkaloid, suppresses NSCLC tumor and cell growth by inhibiting the KIF11-CDK1-CDC25C-Cyclin B1-mediated G2/M pathway and the NF-κB/JNK signaling pathway (44). CDC25C is aberrantly expressed in various tumors, and its high expression significantly correlates with shorter overall survival and advanced pathological stages in NSCLC, consistent with our findings (45).

In this study, glutathione S-transferase Mu 2 (GSTM2) was identified as a protective factor in NSCLC. This is consistent with the findings of Zhang et al., who identified GSTM2 as a protective factor in a LUAD risk model (46). Tang et al. confirmed the protective role of GSTM2 in NSCLC, with its high expression associated with favorable patient survival and significant suppression of tumor growth and metastasis in overexpression animal models (47). In this study, GSTM2 expression was significantly upregulated in PC-9 cells after PYYFD treatment. As a member of the glutathione transferase family, GSTM2 plays important roles in detoxifying or metabolizing various substances including drugs (48). GSTM2 also plays a critical role in protection against oxidative damage and is therefore regarded as an antioxidant gene (49). Nicotinamide mononucleotide was shown to mitigate silicon-induced pulmonary oxidative stress and tissue injury by increasing the expression of antioxidant genes, including GSTM2 (50). These results suggest that GSTM2 may exert anti-NSCLC effects by participating in oxidative stress regulation and detoxification or metabolic processes. However, due to the lack of further experimental evidence, it remains unclear whether GSTM2 directly mediates the anti-NSCLC effects of PYYFD.

BIRC5, also known as survivin, regulates the cell cycle and apoptosis and has long been regarded as a prominent anti-tumor target (51). In NSCLC, BIRC5 has multiple functions, on the one hand, it exerts immune-related effects and promotes tumor progression by inhibiting apoptosis and enhancing malignant cell mitosis and angiogenesis (52). On the other hand, BIRC5 is a prognostic biomarker for NSCLC, and multiple previous studies have confirmed that high BIRC5 expression is closely associated with an aggressive phenotype, drug resistance, and poor prognosis (53). Similarly, our study found that BIRC5 significantly upregulated in NSCLC tumors and identified it as a risk factor. Furthermore, BIRC5 appears to be involved in modulating the NSCLC tumor microenvironment. Previous evidence indicated that BIRC5 expression was inversely associated with NK cells, B cells, and macrophages, whereas it was positively associated with Treg cells (52). This differs slightly from our findings, which showed that BIRC5 was highly expressed only in NK cells. This difference may result from variations in study resolution and sample heterogeneity.

NUF2, also known as cell division cycle-associated protein 1, is expressed in various types of cancer, but not in normal adult tissues, except in the testes (54). NUF2 playing a role in maintaining centromere microtubule stability and regulating the cell cycle (55). An *in vitro* and *in vivo* study showed that berberine inhibits NSCLC cell proliferation and tumor growth by targeting and suppressing the SPC25/NUF2 axis (56). NUF2 is considered a potential prognostic biomarker for NSCLC, and its high expression is significantly associated with poor prognosis and lymph node metastasis in NSCLC patients (54). In our study, NUF2 was identified as a significant risk-associated factor in NSCLC. ACHE encodes acetylcholinesterase, whose classical function is to regulate cholinergic neurotransmission. With increasing research, ACHE has also been shown to exert non-cholinergic functions in cancer, such as influencing apoptosis, regulating inflammation, and modulating cell proliferation and differentiation, thereby participating in tumorigenesis and tumor progression (57). In addition, ACHE has been identified as a therapeutic target for NSCLC in multiple studies. Liu et al. showed that solasonine inhibits A549 cells by downregulating ACHE expression, exerting anti-inflammatory effects, and inducing apoptosis (58). This is similar to our findings, in which ACHE expression decreased after PYYFD treatment. However, this finding requires further experimental validation.

Ephrin receptor B2 (EPHB2) plays dual roles in different tumors, and both our study and previous reports indicate that EPHB2 acts as an oncogenic factor in NSCLC (59). As a member of the Eph family, EPHB2 participates in the cellular communication system and plays key roles in cell adhesion, migration, and tissue morphogenesis (60). A recent study showed that EPHB2 knockout significantly reduced the proliferation and migration of PC-9 cells and that EPHB2 may serve as a marker of M2 macrophage infiltration in LUAD (61). The role of ADH1C in the oxidation of ethanol into the carcinogenic metabolite acetaldehyde varies inconsistently across different tumours (62). Recent studies indicate that high ADH1C expression is associated with better prognosis and longer OS in NSCLC patients (63). In agreement with our results, ADH1C was identified as a protective factor for NSCLC. Evidence from an A549 cell-based study indicated that suppressing ADH1C expression reduced cell proliferation and cisplatin resistance (64). Interestingly, both our study and previous studies have revealed a discrepancy between changes in ADH1C expression levels and its role as a protective factor. One possible explanation is that ADH1C, as a protective factor, reflects the disease status of NSCLC patients rather than acting as a direct mediator of PYYFD. Therefore, further functional studies are required to determine whether ADH1C is a direct functional target of PYYFD.

The collagen type I alpha 1 chain (COL1A1) is an extracellular matrix protein that plays multiple roles in NSCLC. It serves as a potential prognostic marker and therapeutic target involved in tumour cell growth, metastasis and the development of drug resistance (65, 66). Yang et al. found that COL1A1 may be a potential target of quercetin and plays an important role in overcoming carboplatin resistance in ovarian cancer (67). Another molecular docking study demonstrated that COL1A1 and quercetin had a strong binding affinity, with a binding energy of -9.9 kcal/mol (68). Although these results are consistent with our results, direct experimental evidence confirming the interaction between COL1A1 and quercetin is still lacking. Earlier studies have found a strong link between COL1A1 and the tumour microenvironment, as well as immune cell infiltration in NSCLC. The level of COL1A1 expression has been shown to be positively associated with the levels of infiltration by macrophages and neutrophils in LC (69). Studies demonstrate that COL1A1 promotes tumor growth and metastasis by influencing fibroblast migration (70, 71). Cancer-associated fibroblasts (CAFs), as major components of the tumor microenvironment, can influence tumor progression by secreting cytokines such as growth factors and chemokines, thereby regulating inflammation, stroma, and cellular growth and proliferation (72). In NSCLC, CAFs affect patient prognosis through different phenotypes (73). In our study, fibroblasts could be transcriptionally separated into two major states annotated as EMT-like fibroblasts and myofibroblasts. EMT-like Fibroblasts refer to fibroblasts exhibiting epithelial-mesenchymal transition (EMT) characteristics, playing complex and critical roles in NSCLC development (74).

Gene signature-based prognostic risk models have been increasingly developed in recent years. For example, Zhang et al. constructed and validated an NSCLC prognostic model comprising 16 T cell-related genes, which showed a certain degree of predictive robustness (AUC > 0.6) (75). Another study identified eight hypoxia-related TME features that showed significant accuracy in predicting survival in patients with NSCLC (76). Based on prognostic genes, we constructed an NSCLC prognostic risk model and validated its generalizability in two independent external datasets. The results indicated that this model had a certain prognostic predictive ability, but its clinical value was limited (AUCs > 0.6). Simultaneously, risk score and age were identified as independent prognostic factors for predicting NSCLC, leading to the construction of a nomogram model. Nomograms have been widely used for prognostic prediction in cancer patients and are regarded as more effective than conventional TNM staging (77). Therefore, this prognostic risk model remains some distance from translation into clinical practice.

However, this study inevitably has limitations. First, data were sourced from public databases with limited sample size, which may limit the generalizability of the findings. Notably, the scRNA-seq analysis was based on only two tumor samples and two matched normal tissues, which may limit the comprehensive characterization of the immune microenvironment in NSCLC. Furthermore, feature selection and model training both used TCGA-NSCLC, which introduces optimistic bias. Therefore, further validation in larger cohorts is required to confirm the findings of this study. Second, although these findings were preliminarily validated in PC9 cells, this is insufficient to fully elucidate the above observations, and further validation is needed in NSCLC cell lines with different molecular subtypes and in clinical samples. In addition, the findings of this study mainly rely on computational analyses and lack further functional experimental validation. For example, although molecular docking suggested a computationally predicted potential interaction between COL1A1 and quercetin, further molecular interaction experiments are required for validation. In addition, chemical characterization of PYYFD is required. Therefore, experimental validation of these discoveries is essential and will be the focus of our future research.

Overall, we preliminarily explored the potential mechanisms underlying the therapeutic effects of PYYFD in NSCLC. A total of 95 candidate therapeutic targets were identified, and these genes were significantly enriched in multiple immune-related pathways. The prognostic value of these candidate therapeutic targets was further investigated, and eight prognostic genes were ultimately identified, including CDC25C, GSTM2, BIRC5, EPHB2, NUF2, COL1A1, ADH1C, and ACHE. These findings may provide valuable references and insights into the treatment of NSCLC with PYYFD.

## Data Availability

Publicly available datasets were analyzed in this study. This data can be found here: Gene Expression Omnibus (GEO) database (https://www.ncbi.nlm.nih.gov/geo/), and the UCSC Xena platform (https://xenabrowser.net).
