# Supplementary material for: Exploring the potential mechanisms of Pei Yuan Yi Fei decoction for non-small cell lung cancer through network pharmacology and experimental validation
Source: Front Oncol. 2026 Oct 19;16:1938084. doi: 10.3389/fonc.2026.1938084 (PMC13724363; doi:10.3389/fonc.2026.1938084)
Supplement: Supplementary file 1 [file SupplementaryFile1.docx]

**Supplementary Table 1 Baseline clinical characteristics of the training cohort.**

| Variable | Total | vital | | P-value |
| --- | --- | --- | --- | --- |
|  |  | Alive | Dead |  |
| age(year) |  |  |  |  |
| Mean (SD) | 66.3 (±9.3) | 65.8 (±9.2) | 67.2 (±9.5) | 0.01 |
| gender |  |  |  |  |
| FEMALE | 394 (40.0%) | 250 (42.2%) | 144 (36.7%) | 0.097 |
| MALE | 591 (60.0%) | 343 (57.8%) | 248 (63.3%) |  |
| vital_status |  |  |  |  |
| Alive | 593 (60.2%) | 593 (100.0%) | 0 (0.0%) | < 0.001 |
| Dead | 392 (39.8%) | 0 (0.0%) | 392 (100.0%) |  |
| OS(Months) |  |  |  |  |
| Mean (SD) | 941.3 (±935.6) | 1011.0 (±996.9) | 835.9 (±824.6) | < 0.001 |
| pathologic_M |  |  |  |  |
| M0 | 731 (74.7%) | 431 (73.2%) | 300 (77.1%) | < 0.001 |
| M1 | 31 (3.2%) | 11 (1.9%) | 20 (5.1%) |  |
| MX | 216 (22.1%) | 147 (25.0%) | 69 (17.7%) |  |
| pathologic_N |  |  |  |  |
| N0 | 633 (64.3%) | 419 (70.8%) | 214 (54.6%) | < 0.001 |
| N1 | 221 (22.5%) | 112 (18.9%) | 109 (27.8%) |  |
| N2 | 109 (11.1%) | 49 (8.3%) | 60 (15.3%) |  |
| N3 | 7 (0.7%) | 5 (0.8%) | 2 (0.5%) |  |
| NX | 14 (1.4%) | 7 (1.2%) | 7 (1.8%) |  |
| pathologic_T |  |  |  |  |
| T1 | 278 (28.2%) | 192 (32.4%) | 86 (21.9%) | < 0.001 |
| T2 | 549 (55.7%) | 326 (55.0%) | 223 (56.9%) |  |
| T3 | 114 (11.6%) | 57 (9.6%) | 57 (14.5%) |  |
| T4 | 41 (4.2%) | 17 (2.9%) | 24 (6.1%) |  |
| TX | 3 (0.3%) | 1 (0.2%) | 2 (0.5%) |  |
| pathologic_stage |  |  |  |  |
| Stage I | 502 (51.6%) | 340 (58.1%) | 162 (41.8%) | < 0.001 |
| Stage II | 276 (28.4%) | 160 (27.4%) | 116 (29.9%) |  |
| Stage III | 163 (16.8%) | 74 (12.6%) | 89 (22.9%) |  |
| Stage IV | 32 (3.3%) | 11 (1.9%) | 21 (5.4%) |  |

**Supplementary Table 2 Baseline clinical characteristics of the GSE42127.**

| Variable | Total | vital | | P-value |
| --- | --- | --- | --- | --- |
|  |  | Alive | Dead |  |
| age at surgery(years) | | | | |
| Mean (SD) | 66.3 (±9.8) | 64.8 (±9.8) | 69.1 (±9.2) | 0.004 |
| gender | | | | |
| F | 83 (47.2%) | 61 (54.5%) | 22 (34.4%) | 0.012 |
| M | 93 (52.8%) | 51 (45.5%) | 42 (65.6%) |  |
| vital_status | | | | |
| Alive | 112 (63.6%) | 112 (100.0%) | 0 (0.0%) | < 0.001 |
| Dead | 64 (36.4%) | 0 (0.0%) | 64 (100.0%) |  |
| OS(Months) | | | | |
| Mean (SD) | 1518.3 (±970.1) | 1724.5 (±982.9) | 1157.6 (±839.2) | < 0.001 |
| histology | | | | |
| Adenocarcinoma | 133 (75.6%) | 90 (80.4%) | 43 (67.2%) | 0.068 |
| Squamous | 43 (24.4%) | 22 (19.6%) | 21 (32.8%) |  |
| final.pat.stage | | | | |
| IA | 42 (23.9%) | 33 (29.5%) | 9 (14.1%) | 0.067 |
| IB | 70 (39.8%) | 47 (42.0%) | 23 (35.9%) |  |
| IIA | 9 (5.1%) | 5 (4.5%) | 4 (6.2%) |  |
| IIB | 23 (13.1%) | 12 (10.7%) | 11 (17.2%) |  |
| IIIA | 13 (7.4%) | 6 (5.4%) | 7 (10.9%) |  |
| IIIB | 17 (9.7%) | 9 (8.0%) | 8 (12.5%) |  |
| IV | 1 (0.6%) | 0 (0.0%) | 1 (1.6%) |  |
| unknown | 1 (0.6%) | 0 (0.0%) | 1 (1.6%) |  |
| had_adjuvant_chemo | | | | |
| TRUE | 49 (27.8%) | 40 (35.7%) | 9 (14.1%) | 0.003 |
| FALSE | 127 (72.2%) | 72 (64.3%) | 55 (85.9%) |  |

**Supplementary Table 3 Baseline clinical characteristics of the GSE31210.**

| Variable | Total | vital | |  |
| --- | --- | --- | --- | --- |
|  |  | Alive | Dead | P-value |
| age(years) | | | | |
| Mean (SD) | 59.6 (±7.4) | 59.4 (±7.2) | 60.6 (±8.4) | 0.24 |
| gender | | | | |
| female | 121 (53.5%) | 105 (55.0%) | 16 (45.7%) | 0.36 |
| male | 105 (46.5%) | 86 (45.0%) | 19 (54.3%) |  |
| vital_status | | | | |
| Alive | 191 (84.5%) | 191 (100.0%) | 0 (0.0%) | < 0.001 |
| Dead | 35 (15.5%) | 0 (0.0%) | 35 (100.0%) |  |
| OS(Months) | | | | |
| Mean (SD) | 1722.9 (±689.2) | 1848.7 (±634.9) | 1036.5 (±563.2) | < 0.001 |
| pstage iorii | | | | |
| I | 168 (74.3%) | 151 (79.1%) | 17 (48.6%) | < 0.001 |
| II | 58 (25.7%) | 40 (20.9%) | 18 (51.4%) |  |
| pathologic_stage | | | | |
| IA | 114 (50.4%) | 106 (55.5%) | 8 (22.9%) | < 0.001 |
| IB | 54 (23.9%) | 45 (23.6%) | 9 (25.7%) |  |
| II | 58 (25.7%) | 40 (20.9%) | 18 (51.4%) |  |
| relapse | | | | |
| not relapsed | 162 (71.7%) | 160 (83.8%) | 2 (5.7%) | < 0.001 |
| relapsed | 64 (28.3%) | 31 (16.2%) | 33 (94.3%) |  |
| smoking status | | | | |
| Ever-smoker | 111 (49.1%) | 91 (47.6%) | 20 (57.1%) | 0.36 |
| Never-smoker | 115 (50.9%) | 100 (52.4%) | 15 (42.9%) |  |
| MYC | | | | |
| High | 17 (7.5%) | 15 (7.9%) | 2 (5.7%) | 1 |
| Low | 207 (91.6%) | 174 (91.1%) | 33 (94.3%) |  |
| nd | 2 (0.9%) | 2 (1.0%) | 0 (0.0%) |  |
| gene alteration status | | | | |
| ALK-fusion + | 11 (4.9%) | 9 (4.7%) | 2 (5.7%) | 0.12 |
| EGFR mutation + | 127 (56.2%) | 113 (59.2%) | 14 (40.0%) |  |
| EGFR/KRAS/ALK - | 68 (30.1%) | 52 (27.2%) | 16 (45.7%) |  |
| KRAS mutation + | 20 (8.8%) | 17 (8.9%) | 3 (8.6%) |  |

**Supplementary Table 4 Parameters for molecular docking.**

| **Target Name** | **PDB ID** | **protein chain** | **resolution** | **ligand-binding site** | **grid center** | **grid dimensions** | **exhaustiveness** | **number of poses** |
| --- | --- | --- | --- | --- | --- | --- | --- | --- |
| COL1A1 | 7E7D | Trimers: Chains A, B, and C | 3.20 Å | RBD ASN-978, ASN-856 | x=152.116 Å, y=152.157 Å, z=153.57 Å | size_x=100, size_y=98, size_z=88 | 0.375 Å | num_modes=10 |

**Supplementary Table 5 Time-course effects of the PYYFD Solution on cell viability.**

| Concentration (mg/mL) | 24 h (%, mean ± SD) | 48 h (%, mean ± SD) | 72 h (%, mean ± SD) | n |
| --- | --- | --- | --- | --- |
| Vehicle control | 99.44 ± 0.71 | 99.58 ± 0.32 | 97.02 ± 1.24 | 3 |
| Control | 102.63 ± 2.04 | 100.13 ± 0.52 | 94.34 ± 6.03 | 3 |
| 0.1 | 98.80 ± 0.18 | 95.29 ± 1.53 | 78.76 ± 6.45 | 3 |
| 1 | 86.91 ± 1.76 | 93.02 ± 0.53 | 53.15 ± 4.23 | 3 |
| 2 | 84.09 ± 4.11 | 84.86 ± 1.95 | 49.99 ± 3.25 | 3 |
| 4 | 71.65 ± 2.21 | 61.95 ± 0.60 | 33.01 ± 1.81 | 3 |
| 8 | 66.08 ± 3.79 | 51.46 ± 0.47 | 35.70 ± 6.57 | 3 |
| 16 | 57.50 ± 2.22 | 23.88 ± 0.96 | 21.21 ± 6.24 | 3 |

**Supplementary Table 6 The sequences of the primers for RT-qPCR.**

| Gene name | Forword | Reverse |
| --- | --- | --- |
| CDC25C | AACAGGCCAAGACTGAAGCA | GCCCTTCCTGAGCTTTCCTT |
| GSTM2 | CTGGGGTACTGGAACATCCG | CATAATCAGGAGCGTCCCCC |
| EPHB2 | GGCCAAATTGTCAACACGCT | CCACTCGTCCACCGTGTTAA |
| NUF2 | AGCAAAACGGACAAGTCGGT | TGTGCGGCGTTTAACTGTTG |
| ADH1C | GGGTCGGCCTATCTGTTGTT | GGCACCCAACTCTTTAGCCT |
| COL1A1 | AAGAGGCATGTCTGGTTCGG | CAGGAAGGTCAGCTGGATGG |
| BIRC5 | TGCTTCAAGGAGCTGGAAGG | CGCACTTTCTCCGCAGTTTC |
| ACHE | GACTGTGCTGGTGTCCATGA | CCTCTGATCCAGGAGACCCA |
| GAPDH | CACCATCTTCCAGGAGCGAG | AAATGAGCCCCAGCCTTCTC |

**Supplementary Table 8 Univariate Cox analysis results of prognostic genes.**

| id | HR | HR.95L | HR.95H | pvalue |
| --- | --- | --- | --- | --- |
| CDC25C | 1.150472163 | 1.058413158 | 1.250538306 | 0.000987395 |
| GSTM2 | 0.926130288 | 0.876535736 | 0.978530909 | 0.006279157 |
| BIRC5 | 1.095337536 | 1.022032674 | 1.173900158 | 0.009977494 |
| EPHB2 | 1.097537541 | 1.021374794 | 1.179379656 | 0.011201931 |
| NUF2 | 1.086557236 | 1.011505504 | 1.167177661 | 0.023012481 |
| COL1A1 | 1.067633749 | 1.005585715 | 1.133510355 | 0.032169287 |
| ADH1C | 0.968633093 | 0.940389858 | 0.997724574 | 0.034785461 |
| ACHE | 0.949690671 | 0.903634659 | 0.998094043 | 0.041832574 |

**Supplementary Table 9 LASSO analysis results.**

|  | beta | se | HR | HR_95L | HR_95H | z |
| --- | --- | --- | --- | --- | --- | --- |
| CDC25C | 0.096 | 0.099 | 1.100 | 0.907 | 1.335 | 0.967 |
| GSTM2 | -0.074 | 0.033 | 0.929 | 0.871 | 0.991 | -2.245 |
| BIRC5 | 0.050 | 0.092 | 1.052 | 0.877 | 1.261 | 0.544 |
| EPHB2 | 0.107 | 0.046 | 1.113 | 1.016 | 1.219 | 2.303 |
| NUF2 | 0.010 | 0.106 | 1.010 | 0.821 | 1.243 | 0.097 |
| COL1A1 | 0.035 | 0.042 | 1.036 | 0.954 | 1.124 | 0.843 |
| ADH1C | -0.026 | 0.020 | 0.974 | 0.938 | 1.013 | -1.323 |
| ACHE | -0.039 | 0.033 | 0.962 | 0.901 | 1.027 | -1.157 |

Supplementary Figure 1. Preliminary experimental results.

Supplementary Figure 2. Single-cell RNA sequencing quality control. (A): Violin plots of quality control (QC) metrics: genes/cell (nFeature_RNA), transcripts/cell (nCount_RNA), mitochondrial content (percent.mt); (B): Mutated gene distribution (top 10 highlighted); (C): PCA plot of sample clustering (PC1 vs. PC2); (D): Elbow plot for dimensionality selection.

Supplementary Figure 3. Heatmap of fibroblasts in relation to other cells.

Supplementary Figure 4. Original Western blot images. (A) COL1A1; (B) NUF2; (C) BIRC5; (D) GAPDH.

Supplementary Figure 5. Morphological changes in PC-9 cells after treatment with different concentrations of PYYFD for 48 h. (A) Results of the CCK-8. (B-E) PC-9 cells without treatment, and PC-9 cells treated with low-, medium-, and high-concentration PYYFD, respectively.
